# Corticotropin releasing factor alters the functional diversity of accumbal cholinergic interneurons

**DOI:** 10.1152/jn.00348.2023

**Published:** 2024-06-19

**Authors:** Anna E. Ingebretson, Yanaira Alonso-Caraballo, John A. Razidlo, Julia C. Lemos

**Affiliations:** ^1^Department of Neuroscience, University of Minnesota-Twin Cities, Minneapolis, Minnesota, United States; ^2^Medical Discovery Team on Addiction, University of Minnesota, Minneapolis, Minnesota, United States

**Keywords:** cholinergic interneuron, corticotropin-releasing factor, nucleus accumbens

## Abstract

Cholinergic interneurons (ChIs) provide the main source of acetylcholine in the striatum and have emerged as a critical modulator of behavioral flexibility, motivation, and associative learning. In the dorsal striatum (DS), ChIs display heterogeneous firing patterns. Here, we investigated the spontaneous firing patterns of ChIs in the nucleus accumbens (NAc) shell, a region of the ventral striatum. We identified four distinct ChI firing signatures: regular single-spiking, irregular single-spiking, rhythmic bursting, and a mixed-mode pattern composed of bursting activity and regular single spiking. ChIs from females had lower firing rates compared with males and had both a higher proportion of mixed-mode firing patterns and a lower proportion of regular single-spiking neurons compared with males. We further observed that across the estrous cycle, the diestrus phase was characterized by higher proportions of irregular ChI firing patterns compared with other phases. Using pooled data from males and females, we examined how the stress-associated neuropeptide corticotropin releasing factor (CRF) impacts these firing patterns. ChI firing patterns showed differential sensitivity to CRF. This translated into differential ChI sensitivity to CRF across the estrous cycle. Furthermore, CRF shifted the proportion of ChI firing patterns toward more regular spiking activity over bursting patterns. Finally, we found that repeated stressor exposure altered ChI firing patterns and sensitivity to CRF in the NAc core, but not the NAc shell. These findings highlight the heterogeneous nature of ChI firing patterns, which may have implications for accumbal-dependent motivated behaviors.

**NEW & NOTEWORTHY** Cholinergic interneurons (ChIs) within the dorsal and ventral striatum can exert a major influence on network output and motivated behaviors. However, the firing patterns and neuromodulation of ChIs within the ventral striatum, specifically the nucleus accumbens (NAc) shell, are understudied. Here, we report that NAc shell ChIs have heterogeneous ChI firing patterns that are labile and can be modulated by the stress-linked neuropeptide corticotropin releasing factor (CRF) and by the estrous cycle.

## Graphical abstract

**Figure:**
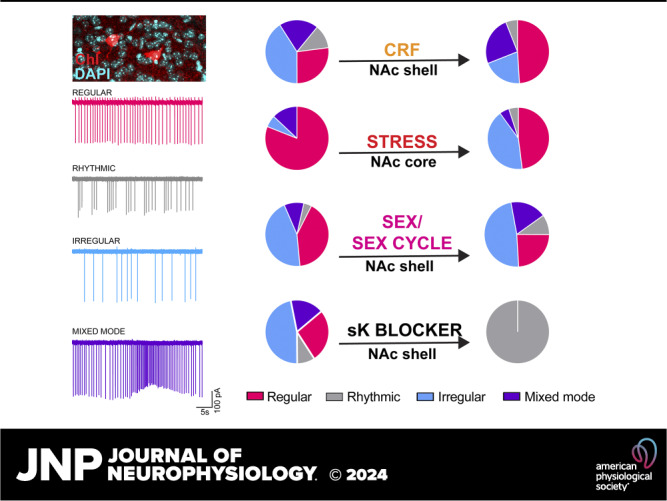

## INTRODUCTION

Cholinergic interneurons (ChIs) provide the main source of acetylcholine modulation of striatal circuit function (1). ChIs make up ∼1–2% of all the neurons within the dorsal and ventral striatum (2–4). However, these large aspiny neurons are highly ramified such that this relatively small number of neurons can serve as a master regulator of striatal output (1, 4). A central functional property of this population of neurons is that they are spontaneously active both in in vivo and ex vivo preparations (1). In the dorsal striatum, ChIs display heterogeneous firing patterns. Bennett and Wilson (5) identified three categories of spontaneously firing ChIs within the dorsal striatum. “Regular,” “irregular,” and “rhythmic bursting” signatures could be distinguished by their differential firing rates and coefficients of variation (CVs) of the interevent interval (IEI) distribution (5). “Regular” ChIs had high firing rates distinguished by regularly occurring single spikes that had narrow, unimodal IEI distributions and low IEI CVs, characteristic of canonical pacemaker firing. “Irregular” ChIs had lower firing rates and higher CVs, indicating greater variability in spike timing. Finally, “rhythmic bursting” ChIs were characterized by a rhythmic firing pattern consisting of bursts of spikes interspersed with pauses in firing activity that yielded a skewed or bimodal IEI distribution with shorter intraburst intervals comprising the mean interval and longer interburst pauses forming the tail of the distribution; this high degree of temporal variability produced low overall firing rates and very high IEI CVs (5, 6). Bennet and Wilson went on to demonstrate that these disparate firing properties were not driven by excitatory or inhibitory synaptic transmission, but by differential intrinsic properties. Specifically, the small calcium-activated potassium channel (sK), which regulates somatic excitability, was identified as a key regulator of firing properties (5–8).

In contrast to the dorsal striatum, the firing properties of different cell types within the nucleus accumbens (NAc) core and shell, including ChIs, remain largely unexplored. It is unknown whether the functional heterogeneity of ChIs in the dorsal striatum is also present in the ventral striatum. Although early studies included both male and female animals, sex and sex hormones as biological variables of interest were not explored. Furthermore, while it has been shown that manipulations that reduce or disrupt normal ChI firing cause depression-like behavior in rodents, the impact of stressor exposure on ChI firing patterns or properties has not been explored (9–11). In addition, the functional relevance of the complex spiking behavior of ChIs has not been fully examined. Understanding the cellular properties of NAc shell ChIs may provide important mechanistic insights into their role in motivated behavior.

Recent work has renewed interest in the cellular properties of ChIs and how heterogeneous firing modes might relate to the functional role of ChIs in motivated behavior. For example, shifts from tonic ChI pacemaker activity to a burst-pause mode in the dorsal striatum have been suggested to encode a salient environmental stimulus that generates an attentional and, perhaps, a motivational shift (4, 12–15). To our knowledge, no in vivo electrophysiology studies have been carried out focusing on ChIs in the NAc. However, both bursts and ramps of ChI population activity are correlated with motivated approach behavior in the NAc (16). Furthermore, ChI population activity within the NAc shell both tracks with and promotes reward learning (17). Although a mechanistic understanding of how these salient environmental stimuli are translated into molecular and cellular signals in the NAc is still unclear, one potential mechanism is through the release of neuropeptides.

Corticotropin releasing factor (CRF) is a stress-associated neuropeptide that is widely distributed and released in the brain and the periphery during periods of high arousal and salience, including novelty (18–21). In the cortex, CRF is released in response to anticipatory cues that signal food delivery (22). In the NAc, CRF is released in response to novelty and facilitates novelty exploration (21). Exogenous application of CRF in the NAc can promote appetitive behaviors and potentiate both dopamine and acetylcholine transmission (21, 23–25). Finally, CRF potentiates ChI firing rate in the NAc and dorsal striatum via activation of CRF type 1 receptors (CRF-R1), cyclic adenosine monophosphate (cAMP), and sK channel activation in male mice (26). Given the role of striatal ChIs in encoding salient stimuli, CRF might shape salience encoding in the NAc via modulation of ChI firing patterns. In this study, we investigated the diversity of spontaneous firing properties in the NAc shell of both male and female mice and across the estrous cycle in female mice. We had previously not characterized ChI firing patterns in the NAc shell, nor had we distinguished NAc core versus shell when examining CRF effects. The NAc core and shell have been shown to have different neuroanatomical and physiological properties, contribute to appetitive behaviors in distinct ways, and respond differentially to stress (27–34). Therefore, in this context, we felt that investigation of these two subregions of the NAc was warranted. Here, we examined if ChIs with different spontaneous firing properties differentially respond to CRF application at different concentrations. During this investigation, we observed four distinct categories of ChIs based on temporal firing patterns. We also found interesting sex differences and sensitivity to CRF based on this categorization. Importantly, we found that CRF shifts the distribution of firing modes toward a more regular tonic firing pattern as opposed to a more rhythmic or bursting mode. Finally, we examined how prior stress history impacts ChI firing patterns in the NAc core and shell and how it may shift CRF sensitivity. These data may provide a cellular mechanism for how salient stimuli shift the circuit-level output of the NAc to facilitate appropriate behavioral responses to the environment.

## MATERIALS AND METHODS

All procedures were performed in accordance with protocols approved by the Institutional Animal Care and Use Committee at the University of Minnesota.

### Animals

Male and female mice (*postnatal day 60*–*180*) were group housed and kept under a 12-h light cycle (630 ON/1830 OFF) with food and water available ad libitum. For all ex vivo electrophysiology experiments, ChAT-IRES-Cre^+/−^ mice [B6N.129S6(B6)-Chattm2(cre)Lowl/J, Jackson Laboratory Stock Number 018957] were crossed with Ai14;tdTomato reporter mice [B6.Cg-Gt(ROSA)26Sortm14(CAG-tdTomato)Hze/J, Jackson Laboratory Stock Number 007914] to easily visualize ChIs. C57BL6/J males were used for in situ hybridization experiments and behavior.

### Estrous Cycle Tracking

Vaginal lavage methods were used to track the estrous cycle of female mice over an 8–10 day period to capture at least two full cycles. We used two different methodologies that yielded the same conclusions. First, 20 µL of sterile saline was rapidly pipetted in and out of the vagina to collect vaginal cells within the solution. The sample was dried, stained with cresyl violet, and assessed under the microscope. Based on the proportions of leukocytes, cornified epithelial cells, and nucleated epithelial cells, as well as the size of the vaginal opening, we classified females as in proestrus, estrus, metestrus, or diestrus (35). Later, we were able to use a 24-well plate and inverted light microscope without cresyl violet staining for higher throughput assessment (36).

### Ex Vivo Electrophysiology

Coronal or sagittal brain slices (240 µm) containing the NAc core and shell were prepared from 8- to 24-wk-old *ChAT-ires-CRE^+/−^;Ai14 tdTomato* mice. Sagittal sections were used when directly comparing NAc core and shell since it is easier to differentiate core/shell boundaries in this preparation, particularly in more lateral slices. Slices were cut in ice-cold cutting solution (in mM): 225 sucrose, 13.9 NaCl, 26.2 NaHCO_3_, 1 NaH_2_PO_4_, 1.25 glucose, 2.5 KCl, 0.1 CaCl_2_, 4.9 MgCl_2_, and 3 kynurenic acid. Slices were maintained in oxygenated artificial cerebrospinal fluid (ACSF) containing (in mM): 124 NaCl, 2.5 KCl, 2.5 CaCl_2_, 1.3 MgCl_2_, 26.2 NaHCO_3_, 1 NaH_2_PO_4_, and 20 glucose (∼310–315 mosmol/kgH_2_O) at room temperature following a 1-h recovery period at 33°C. Cell-attached recordings were made in voltage-clamp mode at 30–33°C to assess firing properties and to measure the effect of CRF on ChI firing frequency without causing “run-down” due to dialyzing the cell with internal solution in a whole cell configuration. For cell-attached recordings, electrodes were filled with filtered ACSF identical to the external solution. A gigaohm seal was achieved, maintained, and monitored throughout recordings. Recordings were conducted for a maximum of 15 min. Cells in which the gigaohm seal had degraded were excluded; seal degradation was determined by rapid changes in holding current past 100 pA. Data were acquired at 5 kHz and filtered at 1 kHz using either a SutterPatch or Multiclamp 700B (Molecular Devices). Data were analyzed using Igor or pClamp (Clampfit, v.10.3).

### Drug Application

2,3-Dioxo-6-nitro-1,2,3,4-tetrahydrobenzo[*f*]quinoxaline-7-sulfonamide (NBQX), 3-((*R*)-2-carboxypiperazin-4-yl)-propyl-1-phosphonic acid (R-CPP), and rat/human CRF were acquired from R & D systems (Tocris) and bath applied to the slice preparation. CRF was bath applied at concentrations of 0, 3, 10, and 100 nM. These concentrations of CRF were based on our previous work (26), where we applied CRF to NAc core slices from males and found that the EC_50_ was 8.6 nM and the maximal concentration was 100 nM (i.e., higher concentrations did not produce a larger effect). The last 3 min of CRF application were averaged to produce a mean response. We chose to use an sK channel blocker (apamin) at a submaximal concentration (150 nM) since, in our experience, maximal concentrations of this blocker can lead to total shutdown of firing and disruption of the gigaohm seal.

### Fluorescent In Situ Hybridization Using RNAScope

Brains were rapidly dissected from male mice and flash frozen in isopentane on dry ice. Brains were kept in a −80°C freezer until they were sectioned. Coronal or sagittal brain slices (16 µm) containing the dorsal striatum (DS) and NAc were thaw mounted onto Superfrost plus slides (Electron Microscopy Sciences) using a Leica CM 1900 cryostat maintained at −20°C. Prior to sectioning, brains were equilibrated in the cryostat for at least 2 h. Slides were cleaned with RNaseZap, a decontamination solution used to prevent mRNA degradation (Invitrogen). Slides were stored at −80°C.

RNAScope in situ hybridization (ISH) was conducted according to the Advanced Cell Diagnostics user manual and as previously reported (26). Briefly, slides were fixed in 10% neutral-buffered formalin for 20 min at 4°C. Slides were washed 2 × 1 min with 1× PBS, before progressive dehydration with 50% ethanol (1 × 5 min), 70% ethanol (1 × 5 min), and 100% ethanol (2 × 5 min). Slides were incubated in 100% ethanol at −20°C overnight. The following day, slides were dried at room temperature (RT) for 10 min. A hydrophobic barrier was drawn around the sections using a hydrophobic pen and allowed to dry for 15 min at RT. Sections were then incubated with Protease Pretreat-4 solution (Advanced Cell Diagnostics) for 20 min at RT. Slides were washed with ddH_2_O (2 × 1 min), before being incubated with the appropriate probes for 2 h at 40°C in the HybEZ oven (Advanced Cell Diagnostics). The following probes were purchased from Advanced Cell Diagnostics: Mm-*Crhr1*-C1 (ACD Cat. No.: 418011), Mm-*Crh*-C1 (ACD Cat. No.: 316091), and Mm-*Chat*-C2 (ACD Cat. No.: 408731-C2). (*Crhr1* is the gene name for cortoticotropin releasing factor receptor type 1; *Crh* is the gene name corticotropin releasing factor, also called corticotropin releasing hormone, CRF; *Chat* is the gene name for choline acetyltransferase). Following incubation with the appropriate probes, slides were subjected to a series of amplification steps at 40°C in the HybEZ oven with 2 × 2 min washes (with agitation) in between each amplification step at RT. Amplification steps were performed as follows: *Amp 1* at 40°C for 30 min; *Amp 2* at 40°C for 15 min; *Amp 3* at 40°C for 30 min; *Amp 4-Alt A* at 40°C for 15 min. A DAPI-containing solution was applied to sections (one slide at a time) at RT for 20 s. Finally, slides were coverslipped using ProLong Gold Antifade mounting media (Invitrogen) and stored at 4°C until imaging on a confocal microscope (Zeiss).

### Image Analysis and Quantification

Sections were imaged using a Zeiss confocal microscope and Zen software. Unique ×20 (5 µm thick) and ×40 images (2-µm thick) were acquired from the NAc of stress-naïve and stress-exposed mice using the same software and hardware settings. The settings were titrated for each specific experimental probe. Quantification was performed using Fiji/ImageJ software. Numbers of DAPI cells were automatically generated using the particle counter function in ImageJ. Numbers of ChAT cells and ChAT+/CRF-R1 or +CRF-positive cells were manually counted using the cell counter function. For both ChAT and DAPI cells, cells were considered positive for the experimental probe if there were >5 particles clustered around (but not in) the cell nucleus. Numbers of puncta per ChAT cell (67–71 unique cells for naïve and stress-exposed mice) were analyzed in a semiautomated fashion. Masks were generated using ChAT as a reference point. Rather than using the outline of the cell itself, we used a uniform circle that was the approximate size of the soma and was kept consistent throughout the analysis. The fluorescent image of the experiment probe, in this case, Mm-Crh1 (ChAT+) or Mm-Crh (DAPI), was converted into a binary image after being thresholded. The thresholding was kept consistent across images. Finally, the mask generated with the cell maker image was combined with the binary image of the experimental probe. This generated an image of only the puncta around the soma of our cells of interest. We then used the particle analyzer function to automatically count the number of puncta.

### Forced Swim Stress

Male mice were moved to a behavioral suite and allowed to acclimate for at least 30 min. The swim stress procedure was performed as previously described (21). Briefly, animals were placed in a 20-cm cylinder filled with 4 L of water maintained at 30 ± 1°C for 15 min on *day 1* and 4 × 6 min on *day 2*, separated by 6-min intervals in their home cages. Animals were returned to their home cages for ∼7 days (6–8 days) or ∼14 days (14–18 days), and then ex vivo slices were prepared. This swim stress procedure has been shown to produce CRF release and lead to long-term behavioral changes (37). We used littermates that were maintained in their home cages and not exposed to stress or stress-exposed animals as our control group.

### Locomotor Behavior

Animals were placed in a large (50 × 50 × 40 cm), novel circular open field for 60 min and video monitored. Noldus Ethovision software (v.14) was used to analyze locomotor data.

### Statistics and Classification

We ran a power analysis to determine sample sizes using G*Power 3.1 based on effect sizes identified previously (26). The power analysis is based on the expected magnitudes and standard deviations of this previous study as well as an α value of 0.05. Notably, our previous study using stress-naïve males had statistically less variability than females or stress-exposed animals; as a result, we had to increase our replicates. Statistical analysis was performed in Prism (GraphPad) and Excel on spiking data extracted from the SutterPatch or Multiclamp 700B platforms. For each cell, analysis was performed on 3 min of stable, continuous recording following a 3-min equilibration period. The same procedure was carried out following wash on and equilibration of CRF. Cells with firing rates less than 0.5 Hz were discarded. The mean firing rate for each cell was calculated as the mean number of spikes per second for the analysis recording section. To calculate the coefficient of variation (CV) of spiking activity for each cell, the interevent interval histogram was extracted from the analysis recording section, and both the mean and the standard deviation were calculated. The CV was then calculated as the standard deviation divided by the mean IEI. Clustering analysis was performed on baseline bursting, frequency, and CV data using R (v.4.3.1) with R Studio. The R cluster package was used to calculate the dissimilarity matrix and perform hierarchical clustering; the R clValid package was used to perform internal cluster validation.

For grouped data, a Kolmogorov–Smirnov test was used to test normality. If one or more groups failed normality, nonparametric analyses were chosen. Two-tailed paired *t* tests, one-way ANOVAs, or one-tailed *t* tests were used when appropriate and stated. For analysis of changes in cell proportions, a χ^2^ test was used with numbers that were rounded to the nearest whole integer. All data are presented as means ± SE. Results were considered significant at an α of 0.05. Trends of *P* < 0.1 were reported.

### Blinding and Randomization

Whenever possible, experimenters were blinded to treatment conditions, especially for stress experiments. Mice were assigned to control or stress conditions randomly. Furthermore, ChIs were assigned a CRF concentration randomly to ensure that results were not biased based on baseline firing rate. As a result, firing rates across a particular group were not significantly different for the CRF concentration range.

## RESULTS

### NAc Shell ChIs Have Four Distinct Firing Modes

**Figure 1.**
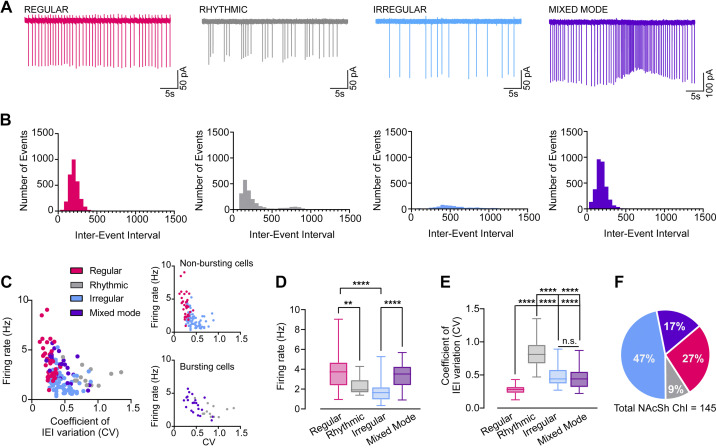
Cholinergic interneurons (ChIs) in the nucleus accumbens (NAc) shell have four distinct firing modes. Example traces of cell-attached recordings (*A*) and corresponding frequency histograms of the interevent interval (*B*) made from regular, rhythmic, irregular, and mixed-mode ChIs recorded in the NAc shell. *C*: firing rate × coefficient of variation (CV) plot for 145 ChIs recorded in NAc shell. ChIs are color coded based on firing mode (regular = magenta, rhythmic = gray, irregular = light blue, mixed mode = purple). Insets separate the plot into nonbursting and bursting cells. There was significant negative correlation between firing rate and CV, *r*^2^ = −0.437, *P* < 0.0001. *D*: summary data of firing rates for regular, rhythmic, irregular, and mixed-mode ChIs. One-way ANOVA with follow-up Tukey’s *t* test. *E*: summary data of CV for interevent interval (IEI) for regular, rhythmic, irregular, and mixed-mode ChIs. Dunn’s post hoc *t* tests were used following a Kruskal–Wallis test. *F*: pie chart of distribution of ChI spontaneous firing pattern from 145 cells recorded in NAc shell of male and female mice. Tukey’s or Dunn’s post hoc *t* test, **P < 0.01, Tukey’s or Dunn’s post hoc *t* test, ****P < 0.00001. *N* = 40 regular cells, 13 rhythmic cells, 68 irregular cells, 24 mixed mode cells.

We sampled a total of 145 ChIs (Cre+) from male and female *ChAT-ires-CRE^+/−^;Ai14 tdTomato* mice. Based on Bennet and Wilson’s (5) observations of regular, irregular, and rhythmic bursting cell types, we classified cells first by observing whether or not the cell displayed bursting activity, defined as at least three burst events consisting of at least three spikes in a burst within a 3-min analysis period. Next, we used the firing rate of the cell and the coefficient of variation (CV) of the cell’s interevent interval distribution, a measure of spike timing variability, to further determine the type of spiking pattern. Based on Bennet and Wilson’s as well as our own observations that firing rate tends to be negatively correlated with CV, we determined that using both metrics provides a more robust assessment of the cell’s spiking signature. Nonbursting cells with regular, single-spiking firing modes and narrow IEI distributions tended to have low CVs of 0.25 or less. Nonbursting irregular cells were characterized by irregularly occurring single spikes, wider IEI distributions, and higher spike timing variability as indicated by CV values of 0.30–0.65. In contrast, rhythmic bursting cells displayed high-frequency bursts of activity followed either by complete pauses in activity or occasional single spikes; these cells usually had skewed or bimodal IEI distributions and the highest CV values typically of 0.70–1.00. However, in addition to these three cell types, we also observed some single-spiking cells that displayed different degrees of spike timing variability as well as intermittent burst activity, with bursting patterns that tended to be longer in duration than bursts in rhythmic bursting cells. As this cell spiking signature appeared to be a mix of both single-spiking and bursting activity, we designated these as “mixed mode” ChIs. Figure 1, *A* and *B*, shows example traces and corresponding IEI frequency histograms of these four different ChI firing patterns. Similar to Bennett and Wilson (5), we observed a significant negative correlation between the firing rate and the IEI CV (*r* = −0.437, *P* < 0.0001), indicating that higher firing rates were associated with lower spike timing variability (Fig. 1*C*). Firing rates tended to range between 1 and 4 Hz, but there were distinct and significant differences across the four ChI types (regular: 3.9 ± 0.4 Hz; rhythmic: 2.3 ± 0.2 Hz; irregular: 1.8 ± 0.1 Hz; mixed mode: 3.3 ± 0.2 Hz, one-way ANOVA, *F*_3,144 _= 24.75, *P* < 0.0001, *n* = 145 cells, see Fig. 1*D* for Tukey’s post hoc *t* tests). Regular ChIs had significantly lower CVs compared with rhythmic or irregular firing patterns, whereas mixed-mode ChIs had significantly higher CVs compared with regular ChIs (regular: 0.27 ± 0.01; rhythmic: 0.84 ± 0.07; irregular: 0.48 ± 0.02; mixed mode: 0.47 ± 0.03, Kruskal–Wallis test, *P* < 0.0001, *n* = 145 cells, see Fig. 1*E* for Dunn’s post hoc comparison). Mixed-mode neurons tended to have higher firing rates compared with irregular single-spiking neurons as well as higher CVs compared with regular neurons due to the presence of bursts. Sorting ChIs based on our evaluation of burst activity, frequency, and CV, we observed that 47% of NAc shell ChIs displayed an irregular firing pattern, 27% had a regular firing pattern, 9% had a rhythmic bursting firing pattern, and 17% had a mixed-mode firing pattern (Fig. 1*F*).

Although this manual evaluation approach relied on both observing each cell’s spiking activity as well as using burst, frequency, and CV metrics to identify ChI spiking types, an unbiased clustering approach was used to validate this method and determine whether ChIs could be naturally grouped based on these spiking metrics. Because quantifying bursting activity is a nontrivial challenge that varies highly across cell type and brain region and is particularly difficult in cell populations with varying spiking signatures, rather than quantifying burst features we instead chose to incorporate bursting as a categorical variable (0 = “Non-bursting”; 1 = “Bursting”) based on whether a cell showed at least three burst events within a 3-min analysis period. We used hierarchical clustering since this approach allows for easily visualizing the relationships between clusters and assessing whether our observations of cell types and manual evaluation method show concordance with this clustering algorithm. We first calculated the dissimilarity matrix, an essential first step in clustering that describes how different, or dissimilar, data points are in space. We used the *daisy* function in the R cluster package to calculate the Gower distance, a distance metric appropriate for scaling mixed variable types, including continuous and categorical (Supplemental Fig. S1, *A* and *B*) (38). We next performed agglomerative hierarchical clustering on the Gower distances using the *hclust* function in the R cluster package. Ward’s linkage method provided the best agglomerative clustering coefficient (0.988 out of 1.000). Adding labels representing our manual evaluation of each cell type to the resulting dendrogram revealed two clear clusters consisting of bursting and nonbursting cell types; bursting cell types were further divided into two groups mainly consisting of rhythmic bursting and mixed-mode cell types, and nonbursting cell types were divided into mostly regular and irregular cell types. These clustering results had a high degree of concordance with our observation of four main cell spiking types divided by bursting activity, suggesting that this is an appropriate grouping approach (Supplemental Fig. S1*A*). Finally, we used an internal clustering validation method to assess the clustering structure of the hierarchical clustering result and compared it against other commonly used clustering methods, including position around medoids (PAMs) and k-means. Using the clValid R package, we calculated two internal cluster validation measures, the Dunn index and the silhouette coefficient. These measures were calculated for hierarchical, PAM, and k-means clustering methods and across cluster solutions of 2–6 possible clusters. We found that hierarchical clustering resulting in two clusters provided the best structure, with a Dunn index of 1.023 and a silhouette coefficient of 0.785 (Supplemental Fig. S1, *C* and *D*). Although this validation measure confirmed that two groups provide the most straightforward clustering solution based on bursting activity, it is apparent from the hierarchical clustering results that these groups can be further divided into regular versus irregular and rhythmic bursting versus mixed mode. This result provides support for the four distinct signatures of spiking activity we observed in the NAc and indicates that our evaluation approach provides a good amount of agreement with an unbiased clustering approach.

### Sex Differences in the Basal Functional Properties of NAc Shell ChIs

**Figure 2.**
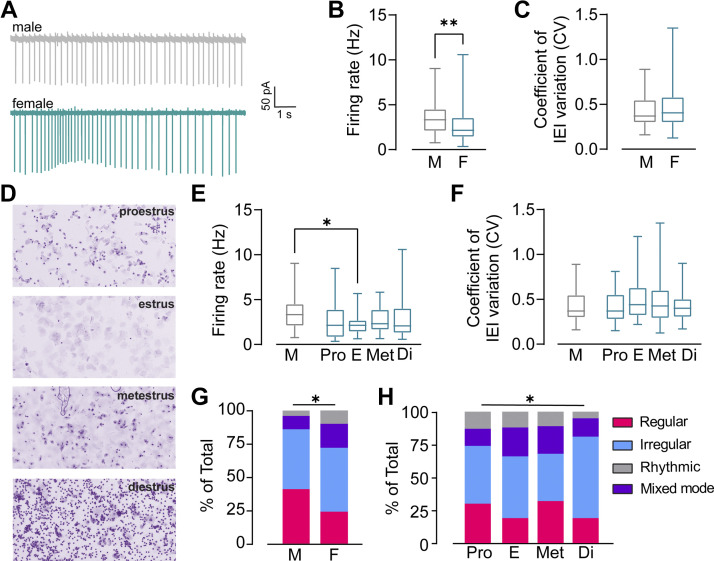
Sex differences in nucleus accumbens (NAc) shell cholinergic interneuron (ChI) firing properties. *A*: example traces of cell-attached recordings made from the NAc shell ChIs in male (*top*) and female (*bottom*) mice. *B*: summary data of firing rates recorded in male and female mice. Mann–Whitney test, **P < 0.01. *C*: summary data of coefficients of variation (CV) of interevent interval (IEI) for ChIs recorded in male and female mice. *D*: example images of vaginal cytology used to classify females into proestrus, estrus, metestrus, and diestrus cycle stages. *E*: summary data of firing rates recorded in the NAc shell ChI from male and female mice across the estrous cycle. Dunn’s post hoc *t* tests were used following a Kruskal–Wallis test: male vs. estrus, *P < 0.05. *F*: summary data of CV of IEI recorded in NAc shell ChI from male and female mice across the estrous cycle. *G* and *H*: distribution of ChI firing mode in male and females (pooled, *left*) across estrous cycle (separated, *right*). χ^2^ test of male vs. female, *P < 0.05; χ^2^ test of female across estrous cycle, *P* < 0.05.

To our knowledge, sex-dependent differences in ChI properties have not been assessed. We analyzed differences in firing rates, IEI CVs, and firing mode proportions between male and female mice and in females across the estrous cycle. On average, males had significantly higher firing rates than females (male: 3.4 ± 0.3 Hz, *n* = 30 cells; female 2.5 ± 0.2 Hz, *n* = 116 cells, Mann–Whitney test, *P* = 0.0089, Fig. 2, *A* and *B*). As described in materials and methods, we tracked the female estrous cycle over an 8- to 10-day period to be confident of estrous cycle stage on the day of euthanasia. A final vaginal lavage was performed prior to euthanasia, and the cycle stage was visualized and noted (Fig. 2*D*). Although firing rates in females appeared lower than males across the estrous cycle, this was only significant in estrus females when the female data was parsed (male: 3.4 ± 0.4 Hz, *n* = 30 cells; female-proestrus: 2.5 ± 0.4 Hz, *n* = 24 cells; female-estrus: 2.2 ± 0.2 Hz, *n* = 32 cells; female-metestrus: 2.6 ± 0.3 Hz, *n* = 20 cells; female-diestrus: 2.7 ± 0.3 Hz, *n* = 37 cells, Kruskal–Wallis test, *P* = 0.0589, Dunnett’s comparison test, male vs. female-estrus, *P* = 0.0286, Fig. 2*E*). Notably, the variance across groups for both firing rates and CVs were significantly different (Bartlett’s test, *P* = 0.0112). On average, males and females did not have significantly different IEI CVs (male: 0.44 ± 0.03, *n* = 30 cells; female 0.45 ± 0.02, *n* = 116 cells, Mann–Whitney, *P* = 0.5357, Fig. 2*C*). Likewise, when comparing the CVs of males and females across the estrous cycle, no significant differences were found (Kruskal–Wallis test, *P* = 0.1435, Fig. 2*F*). We next sorted our previously classified cells by sex and by estrous cycle. Interestingly, we found a significant difference in the proportion of ChI firing signatures between males and females, driven by a higher proportion of mixed-mode firing neurons and a lower proportion of regular firing neurons in female mice (χ^2^ test, *P* = 0.0244, Fig. 2*G*). There were also robust differences in cell firing proportions across the estrous cycle (χ^2^ test, *P* = 0.0134, Fig. 2*H*). Notably, ChIs collected in the estrus phase had higher proportions of mixed-mode and rhythmic bursting firing patterns. ChIs from diestrus females had a very high proportion of irregular firing neurons with very low proportions of regular, mixed-mode, and rhythmic firing patterns, compared with ChIs collected in other cycle stages. Taken together, the data demonstrate differences in ChI firing rates and patterns between males and females.

### Differential CRF Sensitivity Based on ChI Spontaneous Firing Patterns

**Figure 3.**
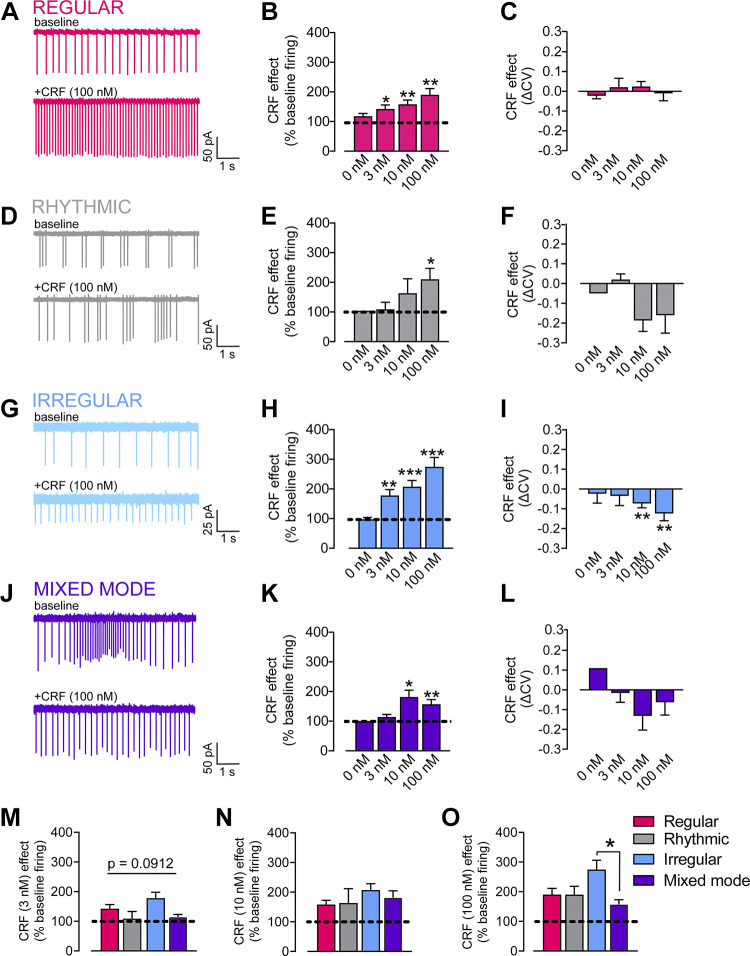
Differential sensitivity to corticotropin releasing factor (CRF) based on cholinergic interneuron (ChI) firing patterns. *A*: example trace of cell-attached recordings prior to and following CRF in from a regular ChI. *B*: summary data of CRF effect on firing rate (% baseline) for 0, 3, 10, 100 nM in regular ChIs. *C*: summary data of CRF effect on coefficients of variation (CV) of interevent interval (IEI) (ΔCV) for 0, 3, 10, 100 nM in regular ChIs. *D*: example trace of cell-attached recordings prior to and following CRF in from a rhythmic ChI. *E*: summary data of CRF effect on firing rate (% baseline) for 0, 3, 10, 100 nM in rhythmic ChIs. *F*: summary data of CRF effect on CV of IEI (ΔCV) for 0, 3, 10, 100 nM in rhythmic ChIs. *G*: example trace of cell-attached recordings prior to and following CRF in from an irregular ChI. *H*: summary data of CRF effect on firing rate (% baseline) for 0, 3, 10, 100 nM in irregular ChIs. *I*: summary data of CRF effect on CV of IEI (ΔCV) for 0, 3, 10, 100 nM in irregular ChIs. *J*: example trace of cell-attached recordings prior to and following CRF in from a mixed-mode ChI. *K*: summary data of CRF effect on firing rate (% baseline) for 0, 3, 10, 100 nM in mixed-mode ChIs. *L*: summary data of CRF effect on CV of IEI (ΔCV) for 0, 3, 10, 100 nM in mixed-mode ChIs. One-sample test vs. 100%, *P < 0.05, one-sample test vs. 100%, **P < 0.01, one-sample test vs. 100%, ***P < 0.0001, one-sample test vs. 100%. *M*: summary data of CRF (3 nM) effect on firing rate (% baseline) for regular, rhythmic, irregular, and mixed-mode ChIs. *N*: summary data of CRF (10 nM) effect on firing rate (% baseline) for regular, rhythmic, irregular, and mixed-mode ChIs. *O*: summary data of CRF (100 nM) effect on firing rate (% baseline) for regular, rhythmic, irregular, and mixed-mode ChIs. One-way ANOVAs, Tukey’s post hoc *t* tests, *P < 0.05, Tukey’s post hoc *t* test, **P < 0.01, Tukey’s post hoc *t* test, ***P < 0.0001.

CRF potentiates ChI firing in the NAc (26). It has been demonstrated that exogenous CRF microinfusion into the NAc promotes locomotor activity and approach behaviors (21, 24, 25, 39). Furthermore, it has been demonstrated that novelty elicits endogenous CRF release into the NAc to promote exploratory behaviors (21). Thus, we speculated that CRF may be a reasonable molecular candidate for how salient stimuli alter the firing properties of ChIs in the NAc. We assessed the ability of CRF to alter the firing rate, CV, and overall firing pattern of NAc shell ChIs collected from male and female mice, segregated into the four classifications based on their baseline firing patterns. We chose CRF concentrations based on previous work done in the NAc core (26). At the maximal concentration (100 nM), CRF significantly increased the firing rate of all four categories of ChI firing patterns (one-sample *t* tests compared with 100% baseline, *n* = 9–20 cells, Fig. 3, *A*, *B*, *D*, *E*, *H*, *I*, *L*, and *K*). However, for 3 nM and 10 nM CRF, the responses varied depending on the basal spontaneous firing pattern of ChIs. CRF (10 nM) increased the firing rate above baseline significantly for ChIs displaying regular, irregular, and mixed-mode firing patterns but not for ChIs showing rhythmic activity (one-sample *t* tests compared with 100% baseline, *n* = 3–24 cells, Fig. 3, *B*, *E*, *L*, and *K*). CRF (3 nM) only increased the firing rate above baseline significantly for regular and irregular firing ChIs (one-sample *t* tests compared with 100% baseline, *n* = 10–24 cells, Fig. 3, *B* and *H*). CRF (10, 100 nM) significantly reduced the IEI CV in irregular firing ChIs only, with no significant effect on regular, rhythmic, or mixed-mode ChIs (one-sample *t* tests compared with 0, *n* = 3–24 cells, Fig. 3, *C*, *G*, *J*, and *M*).

We next directly compared the change in firing rate induced by 3, 10, and 100 nM CRF based on firing pattern. There was a trend for differences in the response at the low concentration (one-way ANOVA, *F*_3,31_ = 2.354, *P* = 0.0912, *n* = 3–15 cells; Fig. 3*M*). Although there were no significant differences at the 10 nM concentration (Fig. 3*N*), at the maximal concentration, CRF differentially potentiated the ChI firing rate based on the basal spontaneous firing pattern (one-way ANOVA, *F*_3,48_ = 3.506, *P* = 0.0222, Tukey’s post hoc *t* test, mixed mode vs. irregular, *P* = 0.0306, *n* = 3–20 cells, Fig. 3*O*). We had previously shown that the CRF response and the baseline firing rate are negatively correlated in ChIs (26). We replicated this finding, demonstrating that the CRF response (percent of baseline firing) was negatively correlated with the baseline firing rate, best fit with a semi-log line (*r*^2^ = −0.60, *P* < 0.001).

### Difference in CRF Sensitivity across the Estrous Cycle

**Figure 4.**
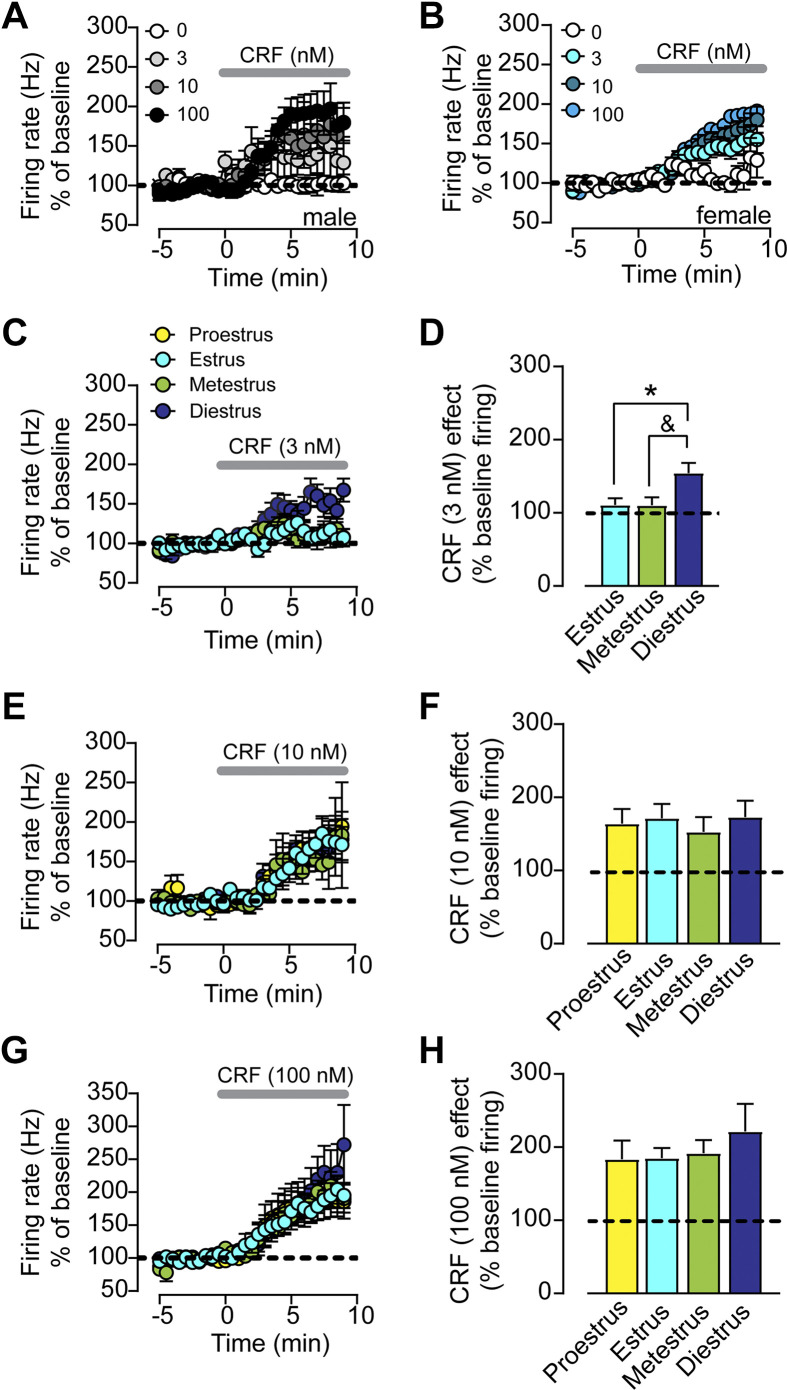
Estrous cycle-dependent difference in sensitivity to a low concentration of corticotropin releasing factor (CRF). *A*: timecourse of normalized firing rate (% baseline) prior to and following bath application of range of concentrations of CRF (0, 3, 10, 100 nM, collected in separate slices) in coronal sections of nucleus accumbens (NAc) shell from male mice. *B*: timecourse of normalized firing rate (% baseline) prior to and following bath application of range of concentrations of CRF (0, 3, 10, 100 nM, collected in separate slices) in coronal sections of NAc shell from female mice. *C*: timecourse of normalized firing rate (% baseline) prior to and following bath application of 3 nM CRF from females in estrus, metestrus, and diestrus. *D*: average maximal CRF (3 nM) response (% baseline) in females in estrus, metestrus, and diestrus. One-way ANOVA, Tukey’s post hoc *t* test, *P < 0.05, Tukey’s post hoc *t* test, &*P* = 0.0565. *E*: timecourse of normalized firing rate (% baseline) prior to and following bath application of 10 nM CRF from females in proestrus, estrus, metestrus, and diestrus. *F*: average maximal CRF (10 nM) response (% baseline) in females in proestrus, estrus, metestrus, and diestrus. *G*: timecourse of normalized firing rate (% baseline) prior to and following bath application of 100 nM CRF from females in proestrus, estrus, metestrus, and diestrus. *H*: average maximal CRF (100 nM) response (% baseline) in females in proestrus, estrus, metestrus, and diestrus.

As there were significant differences in the firing rate and temporal spiking patterns of ChIs between males and females, and between females during different cycles of estrous, we hypothesized that there would be differences in CRF sensitivity given our finding that CRF sensitivity was different depending on the firing pattern. Overall, we did not observe any differences in CRF potentiation of ChI firing rate or change in CVs between males and pooled females (Fig. 4, *A* and *B*). We also did not observe any significant differences in CRF’s impact on the CV across the estrous cycle (data not shown). However, as we would expect given the higher proportion of irregular firing neurons, we did find that a low concentration of CRF (3 nM) potentiated ChI firing rate in diestrus females compared with females in estrus and to less extent in metestrus [timecourse: mixed-effects model, restricted maximum likelihood (REML), *P* > 0.05, fixed effects type III, significant effect of time, *P* < 0.0001, trend for effect of estrous, *P* = 0.0988; one-way ANOVA on the maximal effect, *F*_2,20_ = 4.3539, *P* = 0.0237; Tukey’s post hoc *t* test, diestrus vs. estrus, *P* = 0.0473, diestrus vs. metestrus, *P* = 0.0565, *n* = 6–10 cells, Fig. 4, *C* and *D*]. Proestrus is a very challenging stage of the estrous cycle to capture due to its short duration compared with other stages. As a result, we were not able to collect enough proestrus samples at this concentration, and the cells we did collect that were assigned to 3 nM were all very low firing cells (0.5–1 Hz), making this data uninterpretable. We therefore excluded proestrus from this analysis. We did not find significant differences in CRF sensitivity across the estrous cycle for the 10 nM or 100 nM CRF concentrations (*n* = 8–12 cells for 10 nM and *n* = 7–12 cells for 100 nM; analyses run as aforementioned, *P* values >0.05, Fig. 4, *E*–*H*).

### CRF Alters the ChI Firing Pattern Proportion in the NAc Shell

**Figure 5.**
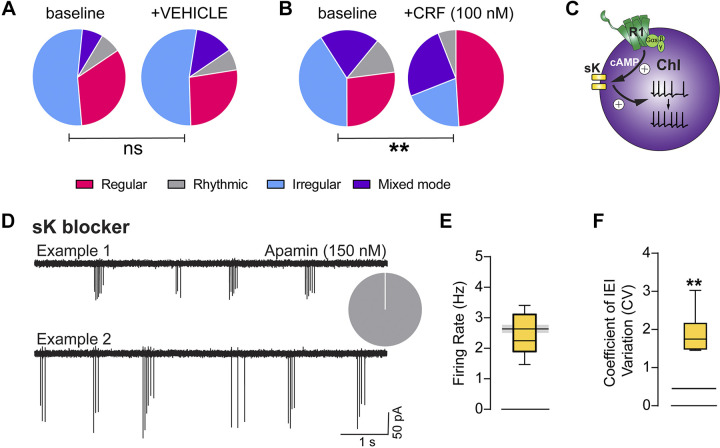
Corticotropin releasing factor (CRF) shifts cholinergic interneuron (ChI) firing properties toward a regular firing pattern. *A*: firing pattern distribution in ChIs prior to (*left*) and following (*right*) vehicle bath application. ns, χ^2^ test. *B*: firing pattern distribution in ChIs prior to (*left*) and following (*right*) CRF (100 nM) bath application. χ^2^ test, **P < 0.01. (regular = magenta, irregular = light blue, rhythmic bursting = grey, mixed mode = purple). *C*: schematic diagram of proposed model for CRF type 1 receptors (CRF-R1) modulation of ChI firing rate. *D*: example traces from cell-attached recordings made from ChIs in the nucleus accumbens (NAc) shell in the presence of the small calcium-activated potassium channel (sK) blocker, Apamin (150 nM). Inset represents the firing pattern distribution observed across ChIs recorded in apamin. *E*: mean firing rate of ChIs recorded in apamin. Means ± SE data from ChIs recorded in drug-free artificial cerebrospinal fluid (ACSF) at a different time are denoted by solid black line, with standard error around the mean in gray. *F*: mean coefficients of variation (CV) of interevent interval (IEI) of ChIs recorded in apamin. Means ± SE data from ChIs recorded in drug-free ACSF at a different time are denoted by solid black line, with standard error around the mean in gray (one-sample *t* tests, **P < 0.01).

It is possible that the firing rate of ChIs could be altered without necessarily affecting the proportions of ChI firing patterns. Thus, we assessed the firing patterns before and following either vehicle or CRF application (100 nM). Similar to the ChI firing pattern proportions assessed from all 145 cells at baseline, the ChIs were composed of 33% regular, 7% rhythmic, 53% irregular, and 7% mixed-mode patterns before vehicle application. There was no significant effect of vehicle application during the same 10-min recording period as was done for CRF application, indicating that the experimental paradigm did not cause artifactual shifts in firing patterns (χ^2^, *P* = 0.4936, *n* = 15 cells, Fig. 5*A*). Before CRF application (100 nM), ChIs were composed of 24% regular, 9% rhythmic, 50% irregular, and 17% mixed-mode patterns. Following CRF (100 nM), these proportions were shifted to 42% regular, 5% rhythmic, 30% irregular, and 23% mixed-mode (χ^2^ test, *P* = 0.0011, *n* = 52 cells, Fig. 5*B*). Our previous work demonstrated that CRF potentiation of ChI firing is dependent on cAMP and sK activation. Furthermore, using a whole cell current-clamp configuration, we demonstrated that CRF reduces firing accommodation, characteristic of ChIs (26). This is schematized in Fig. 5*C*. This G-protein alpha s subunit (Gs) and sK dependency has been attributed to CRF receptor signaling in dopamine neurons in the ventral tegmental area (40) indicating that this may be a common signaling mechanism for CRF modulation, particularly in spontaneously active cells. As CRF alters ChI firing patterns to induce more regular firing activity, we wanted to test whether blockade of sK channels reduces regular firing activity of ChIs in the NAc shell. Attenuation of sK channels with apamin led to a significant shift in the proportions of firing patterns toward entirely rhythmic firing activity (χ^2^ test, *P* < 0.0001) (Fig. 5, *D*–*F*), indicated by a significantly elevated ChI CV of the IEI (one-sample *t* test, *P* = 0.0021), with no change in the overall spiking frequency (one-sample *t* test, *P* = 0.4513, *n* = 6 cells). We compared our apamin recording results to the mean ChI firing rate, CV, and firing pattern distribution of cells recorded in ACSF. Although a within-cell comparison with a higher number of cells and animals would have been ideal, we had limited numbers of ChI reporter animals available to dedicate to this experiment. However, though we only recorded six cells across three animals, the effect is extremely consistent and reliable. Furthermore, this effect was very similar to what has been reported in the dorsal striatum (6). Taken together in the context of previous work, these data indicate that positive neuromodulation of sK channels shifts ChI firing patterns toward more regular firing, whereas attenuation of sK channels promotes bursting or rhythmicity.

### Repeated Stressor Exposure Impacts *Crh* and *Crhr1* mRNA Levels

**Figure 6.**
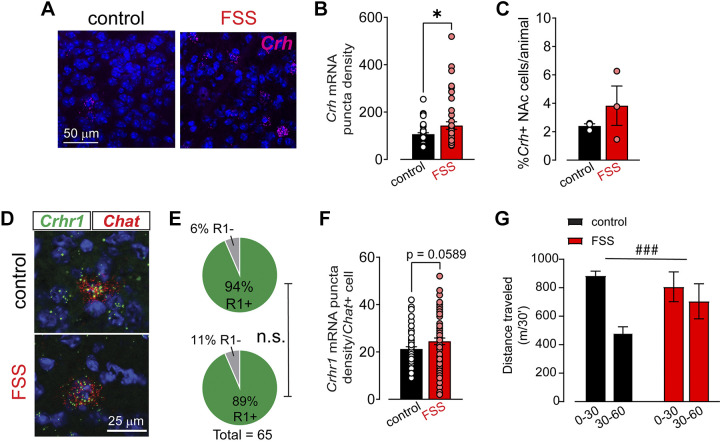
Repeated stressor exposure increases *Crh* mRNA levels in the nucleus accumbens (NAc). *A*: example images of *Crh* mRNA (magenta) with DAPI counterstain in NAc collected from a control male and forced swim stress (FSS) exposed male using fluorescent in situ hybridization techniques. *B*: summary data of number of *Crh* mRNA puncta per DAPI cell collected across three animals per behavioral treatment. 228 *Crh*+ cells for naïve, 330 *Crh*+ cells for FSS. Unpaired *t* test, *P < 0.05. *C*: summary data of %*Crh* + cells in control and FSS exposed mice, *n* = 3 per group. *D*: example images from fluorescent in situ hybridization experiments multiplexing for *Crhr1* mRNA (green) and *Chat* mRNA (red) with DAPI counterstain in NAc collected from a control male and FSS exposed male. *E*: pie charts showing distribution of *Crhr1+/Chat+* and *Crhr1−/Chat+* cells in the NAc of control and stress-exposed male mice. *n* = 74 cells for naïve and 65 cells for FSS. ns, χ^2^ test, *P* > 0.05. *F*: summary data of number of *Crhr1* mRNA puncta per *Chat+* cell collected across three animals per behavioral treatment, unpaired *t* test, *P* = 0.0589. *G*: distance traveled (m/30′) in the first 30 (0–30) and last 30 (30–60) of a 60-min novel open field test conducted in control and FSS-exposed male mice. Two-way ANOVA, time × stress treatment interaction, ###*P* < 0.001.

We next sought to determine whether a behavioral treatment that potentially elevates CRF levels in the NAc, possibly emulating CRF bath application, would shift the firing pattern distribution of ChIs toward a larger proportion of regular firing ChIs. Although it is very difficult to assess CRF release directly, a group of CRF-containing neurons within the NAc has been shown to release CRF onto surrounding ChIs (41). Importantly, using reliable RNAScope methodologies allowed us to assess changes in *Crh* mRNA levels within the NAc following repeated swim stress compared with control animals. We euthanized male mice and flash froze their brains 7 days after the final swim stress exposure. For these stress experiments as well as the ones described later, we chose to use males as we had just established that females have varying ChI firing properties and patterns across baseline as well as varying CRF sensitivity in the stress-naïve state. We found that *Crh* mRNA total puncta was higher in the NAc of stress-exposed animals compared with unperturbed animals [control: 106.7 ± 5.8; forced swim stress (FSS): 143.1 ± 15.95, *t* test, *P* = 0.03, *n* = 44–46 unique images from 3 animals each, Fig. 6, *A* and *B*]. This did not translate into a significant change in the percent of *Crh*+ cells in the NAc (unpaired *t* test, *P* = 0.3655, *n* = 3 animals each, Fig. 6*C*). We also quantified the number of CRF-R1+ ChIs and puncta per ChI from the same animals by multiplexing for *Chat* and* Crhr1* mRNA. We found no significant differences in the number of puncta per CIN cell, though there was a trend for an increase in *Crhr1* puncta in stress-exposed mice, which was not significant (naïve—67 unique ChIs: 21 ± 1 puncta; stress—71 unique ChIs: 25 ± 1.5, *P* = 0.0589, Fig. 6. *D* and *F*) or percent of CRF-R1+ ChIs (χ^2^ test, *P* > 0.05, *n* = 3 animals each, Fig. 6*E*). Previous work demonstrated that CRF microinfusion into the NAc increases locomotor behavior, and novelty exposure triggers CRF release in the NAc (21, 39). We have previously shown that females have an elevated locomotor response to a novel environment compared with males (42). Here, as proof of concept, we tested whether repeated swim stress, which led to elevations in NAc *Crh* levels, impacted novelty-induced locomotor behavior. We found that forced swim stress (FSS) reduced locomotor habituation to a novel environment compared with control conditions (two-way RM ANOVA, time × stress treatment interaction, *F*_1,8 _= 46.47, *P* = 0.0001, *n* = 5 animals each, Fig. 6*G*). This finding provides evidence that stress can alter NAc CRF-dependent behaviors.

### Repeated Stressor Exposure Shifts ChI Firing Distribution and CRF Sensitivity in the NAc Core but Not the NAc Shell

We next assessed the impact of stressor exposure on the firing properties and patterns in the NAc core and the NAc shell as well as assessed stress-induced changes in CRF sensitivity (3, 10, 100 nM). We chose to examine effects at ∼1 wk (6–8 days) and 2 wk (14–18 days) poststressor exposure to determine the persistence of the effects. Again, we chose to use males for these experiments to avoid the additional complexity of estrous-driven changes shown earlier. Furthermore, we chose to use sagittal sections for these experiments since, in our experience, distinguishing core and shell across the medial-lateral axis is more reliable in sagittal sections compared with coronal sections. We confirmed that the plane of section did not significantly affect the firing rate, CV, or firing patterns (Supplemental Fig. S2). We first noted striking differences in the proportions of ChI firing patterns between NAc core and NAc shell in control animals. Although the firing rate was not different between shell and core regions, ChIs of the NAc shell had significantly higher IEI CVs than NAc core ChIs (firing rate: NAc core control: 3.4 ± 0.3 Hz, NAc shell control: 3.4 ± 0.3 Hz, unpaired *t* test, *P* = 0.8292; CV: NAc core: 0.23 ± 0.02, NAc shell control: 0.38 ± 0.03, unpaired *t* test, *P* = 0.0005, *n* = 16–23 cells, Supplemental Fig. S3). This was due to the predominance of regular firing neurons in the NAc core, with only small percentages of mixed-mode, irregular, or rhythmic firing neurons.

**Figure 7.**
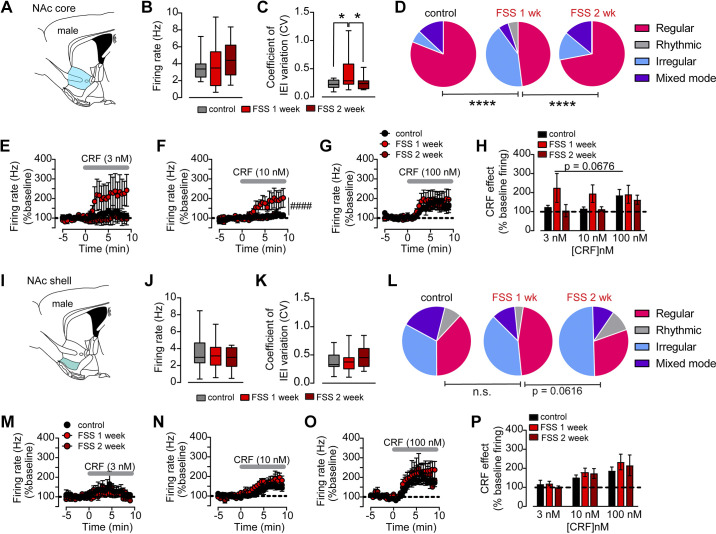
Repeated stressor exposure impacts cholinergic interneuron (ChI) firing pattern distribution and corticotropin releasing factor (CRF) sensitivity in the nucleus accumbens (NAc). *A*: schematic of NAc core region in a sagittal section. *B*: mean baseline firing rate of ChIs recorded from the NAc core of control, forced swim stress (FSS)-1 wk incubation, FSS-2 wk incubation male mice. *C*: mean baseline coefficients of variation (CV) of interevent interval (IEI) of ChIs recorded from the NAc core of control, FSS-1 wk incubation, FSS-2 wk incubation male mice. One-way ANOVA, *Tukey’s post hoc *t* tests, *P* values <0.05. *D*: firing pattern distribution of ChIs recorded from the NAc core of control, FSS-1 wk incubation, FSS-2 wk incubation male mice. ****χ^2^ tests, *P* < 0.0001. (regular = magenta, irregular = light blue, rhythmic bursting = gray, mixed mode = purple). *E*: timecourse of normalized firing rate (% baseline) prior to and following bath application of 3 nM CRF in ChIs recorded from the NAc core of control, FSS-1 wk incubation, FSS-2 wk incubation male mice. *F*: timecourse of normalized firing rate (% baseline) prior to and following bath application of 10 nM CRF in ChIs recorded from the NAc core of control, FSS-1 wk incubation, FSS-2 wk incubation male mice, two-way RM ANOVA, ####time × stress treatment interaction, *P* < 0.0001. *G*: timecourse of normalized firing rate (% baseline) prior to and following bath application of 100 nM CRF in ChIs recorded from the NAc core of control, FSS-1 wk incubation, FSS-2 wk incubation male mice. *H*: average maximal CRF (3, 10, 100 nM) response (% baseline) in NAc core of control, FSS-1 wk incubation, FSS-2 wk incubation male mice. *I*: schematic of NAc shell region in a sagittal section. *J*: mean baseline firing rate of ChIs recorded from the NAc shell of control, FSS-1 wk incubation, FSS-2 wk incubation male mice. *K*: mean baseline CV of IEI of ChIs recorded from the NAc shell of control, FSS-1 wk incubation, FSS-2 wk incubation male mice. *L*: firing pattern distribution of ChIs recorded from the NAc shell of control, FSS-1 wk incubation, FSS-2 wk incubation male mice. (regular = magenta, irregular = light blue, rhythmic bursting = gray, mixed mode = purple). *M*: timecourse of normalized firing rate (% baseline) prior to and following bath application of 3 nM CRF in ChIs recorded from the NAc shell of control, FSS-1 wk incubation, FSS-2 wk incubation male mice. *N*: timecourse of normalized firing rate (% baseline) prior to and following bath application of 10 nM CRF in ChIs recorded from the NAc shell of control, FSS-1 wk incubation, FSS-2 wk incubation male mice. *O*: timecourse of normalized firing rate (% baseline) prior to and following bath application of 100 nM CRF in ChIs recorded from the NAc shell of control, FSS-1 wk incubation, FSS-2 wk incubation male mice. *P*: average maximal CRF (3,10,100 nM) response (% baseline) NAc shell of control, FSS-1 wk incubation, FSS-2 wk incubation male mice.

Strikingly, repeated stressor exposure increased the IEI CV and increased the heterogeneity of ChI firing patterns recorded in the NAc core following 1 wk of incubation after stress. This was fully reversed 2 wk poststressor exposure (CV: control: 0.22 ± 0.02, FSS-1 wk: 0.43 ± 0.06, FSS-2 wk: 0.25 ± 0.03, one-way ANOVA, *F*_2,46 _= 5.682, *P* = 0.0062; proportions of firing patterns: control: 81% regular, 6% irregular, 13% mixed mode, 0% rhythmic; FSS-1 wk: 48% regular, 42% irregular, 5% mixed mode, 5% rhythmic; FSS-2 wk: 72% regular, 14% irregular, 14% mixed mode, 0% rhythmic, χ^2^ tests, *P* values < 0.0001, *n* = 14–19 cells, Fig. 7, *A*–*D*). No changes in the firing rate were observed (control: 3.4 ± 0.3 Hz, FSS-1 wk: 3.9 ± 0.7 Hz, FSS-2 wk: 4.5 ± 0.6 Hz, one-way ANOVA, *F*_2,46 _= 0.9145, *P* = 0.4086).

In contrast, repeated stressor exposure had no significant effect on the firing rate, CV, or firing pattern proportions in the NAc shell, disproving our initial hypothesis that stressor exposure may replicate the effects of acute CRF bath application (firing rate: control: 3.5 ± 0.4 Hz, FSS-1 wk: 3.2 ± 0.3 Hz, FSS-2 wk: 2.9 ± 0.4 Hz, one-way ANOVA, *F*_2,58 _= 0.6266, *P* = 0.5379; CV: control: 0.38 ± 0.03, FSS-1 wk: 0.37 ± 0.03, FSS-2 wk: 0.48 ± 0.07, one-way ANOVA, *F*_2,58 _= 1.632, *P* = 0.2045; pattern distribution: control: 38% regular, 33% irregular, 21% mixed mode, 8% rhythmic; FSS-1 wk: 46% regular, 39% irregular, 11% mixed mode, 4% rhythmic; FSS-2 wk: 30% regular, 50% irregular, 10% mixed mode,10% rhythmic, χ^2^ tests, control vs. FSS-1 wk, *P* = 0.1260, FSS-1 wk vs. FSS-2 wk, *P* = 0.0616, *n* = 10–28 cells, Fig. 7, *I*–*L*).

Consistent with our overall conclusion that irregularly firing ChIs are more sensitive to CRF, we observed differential responses to CRF between the NAc core and shell of control animals (Supplemental Fig. S3) and within the NAc core dataset when comparing control animals to FSS-1 wk and FSS 2-wk animals, particularly at the 10 nM concentration (two-way RM ANOVA, time × treatment interaction, *F*_29.319 _= 2.816, *P* < 0.0001, *n* = 4–7 cells, Fig. 7, *E*–*H*). We did not see any differences in the CRF response for 3 or 100 nM concentrations. We analyzed the average CRF effect in the last 3 min of the experiment for each condition; although there was a trend for a main effect of stress treatment, this was not significant, likely due to the lack of effect at 100 nM and variability in the response at 3 nM (two-way ANOVA, main effect of stress treatment, *F*_2,35 _= 2.912, *P* = 0.0676, *n* = 3–8 cells, Fig. 7, *E* and *H*). In contrast to the NAc core, there were no appreciable differences in the CRF potentiation of ChI firing for any concentration we tested when either looking at the timecourse or average response (same analyses as earlier, *P* values >0.05, *n* = 2–10 cells, Fig. 7, *M*–*P*).

## DISCUSSION

In this study, we characterized the spontaneous firing properties of ChIs within the NAc shell of male and female mice. Previous foundational work described three modes of spontaneous firing patterns in dorsal striatal ChIs in 3- to 4-wk-old rats: regular, rhythmic bursting, and irregular (5). Here, we have extended this work to demonstrate that these patterns are conserved across species, sex, striatal regions, and well into adulthood using an ex vivo slice preparation. However, we identified a novel fourth category of ChIs that is quantitatively and qualitatively distinct from the other three. This fourth mixed-mode group has canonical tonic firing properties like regular or irregular ChIs with intermittent bursting-like rhythmic activity overlaid onto the tonic firing patterns. We further characterized the effect of CRF on the four types of ChI firing patterns and discovered differential sensitivity to CRF, with irregular ChIs potentiating their firing at the lowest and highest concentration of CRF compared with the other groups along with a marked reduction in the CV IEI. A key insight of this study is that acute CRF application shifts the ChI firing pattern distribution toward more regular firing. We show for the first time that sex and estrous cycle impact the ChI firing rate and proportion of ChI firing patterns, and that there are estrous cycle-dependent differences in CRF sensitivity. Finally, we show that repeated stressor exposure increases the heterogeneity of firing pattern proportions in the NAc core and heightens CRF sensitivity while having no impact on the NAc shell. This work is among the first characterizing the firing modes of ChIs in the NAc shell, which may greatly add to our understanding of how these interneurons function on a fundamental level.

Based on previous literature, these spontaneous firing patterns are intrinsic to the cell as they cannot be altered by blockade of glutamatergic or GABAergic synaptic transmission (5). The difference in firing properties is likely due to a difference in ion channel expression and conductance that is highly dependent on different potassium channel conductances. We and others have shown that attenuation of sK channels can cause profound changes in the CV IEI and in the relative proportions of ChI firing patterns (6, 8). For example, attenuation or blockade of sK channels produces a large shift in the CV and firing pattern distribution toward entirely rhythmic bursting cells. These manipulations provide some insight into how endogenous firing modes may be generated and altered. Importantly, Bennett and Wilson (5) demonstrated that these same spontaneous firing patterns are apparent in the intact animal using extracellular recordings, suggesting that differences in firing motifs may impact behavioral function (5). Without having access to the original data, it is challenging to know for certain why Bennett and Wilson (5) did not detect the mixed-mode ChI type. Based on their reports and our previous work, ChIs recorded in the dorsal striatum tend to have lower firing rates than ChIs in the NAc core or shell (5, 26), which might make it more difficult to distinguish irregular and mixed-mode ChIs. It is also possible that NAc shell ChIs have a different composition of ion channels or sK subtypes within the sK channel family that lead to the emergence of this fourth type of ChI firing activity. A potential future direction could entail using a method such as PatchSeq to connect ion channel composition and expression for each cell to their firing pattern.

### Neuromodulation and Plasticity of Ion Channel Function

Here, we report two key findings. First, irregular firing ChIs, characterized by their low firing rate, high CV, and lack of bursting, are most responsive to CRF. Second, high concentrations of CRF are able to shift the firing pattern distribution toward more regular firing. CRF exerts its actions on cell excitability via sK channel enhancement in ChIs (26); in ChIs, it does so by reducing spike accommodation. High sK channel activation gives rise to the medium-duration afterhyperpolarization potential (mAHP), which allows for regular single-spiking activity, whereas low sK channel activity is necessary for bursting (7). Based on both Lemos et al. (26) and Wilson and Goldberg (43), CRF increases sK channel activity in rhythmic bursting and irregular cell types to promote pacemaker activity and prevent bursting activity. High baseline sK activation in regular spiking neurons could occlude CRF effects, particularly at lower concentrations, because regular spiking neurons are already highly active. Though we did not find any studies assessing changes in sK activation across the estrous cycle, changes in the function of other potassium channels across the estrous cycle have been reported. In both GnRH neurons of the medial preoptic area and CRF neurons within the paraventricular nucleus of the hypothalamus, *I*_A_ channel conductance is elevated during estrus and diestrus compared with proestrus (44, 45). Regulation of potassium channel conductances may be a conserved mechanism in which the ovarian hormonal cycle can induce transient changes in the excitability of several cell types across the brain.

### Plasticity in ChI Firing Pattern and CRF Modulation Driven by Sex and Estrous Cycle

Here, we found that NAc shell ChI firing rates and firing patterns differ between male and female mice. Although this difference in ChIs is novel, sex differences in the firing rate of other tonically active neurons such as dopamine neurons have been observed (46). This finding indicates to us that there is state-dependent plasticity in spontaneous firing patterns in NAc shell ChIs. Differences in firing properties across the estrous cycle have also been observed in a number of cell types within the mesocorticolimbic pathway. Estrous cycle-driven changes have also been observed in dopamine neuron firing properties (46, 47). Furthermore, in the cortex, there is a pronounced difference in the firing rate and firing pattern of fast-spiking interneurons (FSIs) recorded in awake behaving nonestrus and estrus females. (48). We posit that different modes of firing across estrous alter the network within the brain region to be more or less responsive to environmental stimuli. For example, in the cortex, these FSIs are only responsive to social touch in nonestrus females, not in estrus females (48).

Differences in CRF responsivity across estrous also have been observed; however, these have often been in the opposite direction to our own observations (49, 50). For example, intracerebroventricular administration of CRF increases grooming behavior more in proestrus females compared with diestrus females or males (50). Here, we found that a low concentration of CRF (3 nM) can potentiate ChI firing in slices taken from diestrus females, but not in estrus females. Novelty has been shown to trigger CRF release in the NAc (21). Differences in CRF sensitivity across the estrous cycle, particularly low concentrations CRF that may encode novelty or salience, but not necessarily stressful/alarming stimuli, may reflect the need to allocate motivational resources differentially throughout the estrous cycle. For example, in diestrus, when reproductive drive is low, females may engage with other environmental stimuli more than during estrus, when reproductive drive is high. Although this is speculative, it has been shown that CRF microinfusion into the NAc shell (but not core) increases locomotor behavior (39) and that females in diestrus show enhanced locomotor responses to a novel environment compared with females in estrus (51, 52).

### Stress-Induced Changes in ChI Firing Properties in the NAc Core, but Not NAc Shell

In this study, we found a profound difference in ChI firing properties and firing patterns between the NAc core and shell of male mice. Under control conditions, ChIs in the NAc core displayed a more homogeneous firing pattern than the NAc shell since regular firing patterns predominated. Repeated stressor exposure transiently shifted NAc core ChI firing patterns (reflected by a change in CV) to resemble the NAc shell (i.e., more heterogeneous, with multiple patterns that dominate including irregular, mixed-mode, and rhythmic neurons). Over time, the firing pattern proportions and CV reverted to the control condition. In contrast, the NAc shell, with cells displaying more heterogeneous firing patterns, was not affected by repeated stressor exposure. Again, consistent with our conclusion that irregular ChI neurons are more responsive to CRF, conditions in which more irregular neurons were part of the ChI firing pattern distribution were associated with greater responsivity to CRF. Specifically, in control mice, NAc shell ChIs had higher sensitivity to CRF (10 nM) than NAc core; comparing control NAc core ChIs with FSS-1 wk NAc core ChIs revealed a similar enhancement of CRF potentiation of ChI firing. This effect is possibly a combination of a change in the proportions of different firing patterns and modest upregulation of CRF-R1 in ChIs. We propose that repeated stressor exposure transiently creates a window in which NAc core ChIs may be more responsive to salient stimuli in the environment that are encoded by CRF. This may manifest as behaviors such as reduced locomotor habituation to novel environment as seen in Fig. 6.

### Limitations

A caveat to our clustering method is the limited information that can be extracted from cell-attached recordings compared with whole cell current-clamp recordings. Future studies should combine the two methods to refine our understanding of ChI firing heterogeneity. Rhythmic bursting cells were sparse in number and were also more challenging to hold at a gigaohm seal through the experiment. This made it challenging to accumulate robust sample sizes for all concentrations of CRF. Thus, we had a significant amount of variability at lower CRF concentrations. Likewise, it was very difficult to capture females in the proestrus and metestrus phases of the estrous cycle since those stages are much shorter than diestrus and estrus. As such, our sample sizes are smaller for those cycle stages. However, considering the difference in the proportions of ChI firing patterns across all four stages, we felt it was important not to pool data into high and low estradiol groups, for example. We can only speculate about what these cellular observations mean for the behaving animal. However, we hope that these ex vivo findings will trigger a new set of testable hypotheses for in vivo studies.

## DATA AVAILABILITY

Data will be made available upon reasonable request.

## SUPPLEMENTAL MATERIAL

Supplemental Figs. S1–S3: https://github.com/jlemosumn/Ingebretson-JN-Supplemental-Material.

## GRANTS

This study was funded by K99/R00 Pathway to Independence Award MH109627 (to J.C.L.) and NIMH BRAINS R01 Grant MH122749.

## DISCLOSURES

No conflicts of interest, financial or otherwise, are declared by the authors.

## AUTHOR CONTRIBUTIONS

A.E.I. and J.C.L. conceived and designed research; A.E.I., Y.A.-C., J.A.R., and J.C.L. performed experiments; A.E.I. and J.C.L. analyzed data; A.E.I. and J.C.L. interpreted results of experiments; A.E.I. and J.C.L. prepared figures; A.E.I. and J.C.L. drafted manuscript; A.E.I. and J.C.L. edited and revised manuscript; J.C.L. approved final version of manuscript.

## References

[1] Gonzales KK, Smith Y. Cholinergic interneurons in the dorsal and ventral striatum: anatomical and functional considerations in normal and diseased conditions. Ann N Y Acad Sci 1349: 1–45, 2015. doi:10.1111/nyas.12762. 25876458 PMC4564338

[2] Bolam JP, Wainer BH, Smith AD. Characterization of cholinergic neurons in the rat neostriatum. A combination of choline acetyltransferase immunocytochemistry, Golgi-impregnation and electron microscopy. Neuroscience 12: 711–718, 1984. doi:10.1016/0306-4522(84)90165-9. 6382048

[3] Bonsi P, Cuomo D, Martella G, Madeo G, Schirinzi T, Puglisi F, Ponterio G, Pisani A. Centrality of striatal cholinergic transmission in Basal Ganglia function. Front Neuroanat 5: 6, 2011. doi:10.3389/fnana.2011.00006. 21344017 PMC3036975

[4] Nosaka D, Wickens JR. Striatal cholinergic signaling in time and space. Molecules 27: 1202, 2022. doi:10.3390/molecules27041202. 35208986 PMC8878708

[5] Bennett BD, Wilson CJ. Spontaneous activity of neostriatal cholinergic interneurons in vitro. J Neurosci 19: 5586–5596, 1999. doi:10.1523/JNEUROSCI.19-13-05586.1999. 10377365 PMC6782311

[6] Bennett BD, Callaway JC, Wilson CJ. Intrinsic membrane properties underlying spontaneous tonic firing in neostriatal cholinergic interneurons. J Neurosci 20: 8493–8503, 2000. doi:10.1523/JNEUROSCI.20-22-08493.2000. 11069957 PMC6773196

[7] Goldberg JA, Wilson CJ. Control of spontaneous firing patterns by the selective coupling of calcium currents to calcium-activated potassium currents in striatal cholinergic interneurons. J Neurosci 25: 10230–10238, 2005. doi:10.1523/JNEUROSCI.2734-05.2005. 16267230 PMC1343481

[8] Wilson CJ. The mechanism of intrinsic amplification of hyperpolarizations and spontaneous bursting in striatal cholinergic interneurons. Neuron 45: 575–585, 2005. doi:10.1016/j.neuron.2004.12.053. 15721243

[9] Cheng J, Umschweif G, Leung J, Sagi Y, Greengard P. HCN2 channels in cholinergic interneurons of nucleus accumbens shell regulate depressive behaviors. Neuron 101: 662–672 e665, 2019. doi:10.1016/j.neuron.2018.12.018. 30638901

[10] Hanada Y, Kawahara Y, Ohnishi YN, Shuto T, Kuroiwa M, Sotogaku N, Greengard P, Sagi Y, Nishi A. p11 in cholinergic interneurons of the nucleus accumbens is essential for dopamine responses to rewarding stimuli. eNeuro 5: ENEURO.0332-18.2018, 2018 [Erratum in eNeuro 5: ENEURO.0493-18.2018, 2018]. doi:10.1523/ENEURO.0332-18.2018. 30417079 PMC6223111

[11] Warner-Schmidt JL, Schmidt EF, Marshall JJ, Rubin AJ, Arango-Lievano M, Kaplitt MG, Ibanez-Tallon I, Heintz N, Greengard P. Cholinergic interneurons in the nucleus accumbens regulate depression-like behavior. Proc Natl Acad Sci USA 109: 11360–11365, 2012. doi:10.1073/pnas.1209293109. 22733786 PMC3396525

[12] Ding JB, Guzman JN, Peterson JD, Goldberg JA, Surmeier DJ. Thalamic gating of corticostriatal signaling by cholinergic interneurons. Neuron 67: 294–307, 2010. doi:10.1016/j.neuron.2010.06.017. 20670836 PMC4085694

[13] Krok AC, Maltese M, Mistry P, Miao X, Li Y, Tritsch NX. Intrinsic dopamine and acetylcholine dynamics in the striatum of mice. Nature. 621: 543–549, 2023. doi:10.1038/s41586-023-05995-9. 37558873 PMC11577287

[14] Zhang YF, Cragg SJ. Pauses in striatal cholinergic interneurons: what is revealed by their common themes and variations? Front Syst Neurosci 11: 80, 2017. doi:10.3389/fnsys.2017.00080. 29163075 PMC5670143

[15] Zucca S, Zucca A, Nakano T, Aoki S, Wickens J. Pauses in cholinergic interneuron firing exert an inhibitory control on striatal output in vivo. eLife 7: e32510, 2018. doi:10.7554/eLife.32510. 29578407 PMC5869016

[16] Mohebi A, Collins VL, Berke JD. Accumbens cholinergic interneurons dynamically promote dopamine release and enable motivation. eLlife 12: e85011, 2023. doi:10.7554/eLife.85011. PMC10259987 37272423

[17] Al-Hasani R, Gowrishankar R, Schmitz GP, Pedersen CE, Marcus DJ, Shirley SE, Hobbs TE, Elerding AJ, Renaud SJ, Jing M, Li Y, Alvarez VA, Lemos JC, Bruchas MR. Ventral tegmental area GABAergic inhibition of cholinergic interneurons in the ventral nucleus accumbens shell promotes reward reinforcement. Nat Neurosci 24: 1414–1428, 2021 [Erratum in Nat Neurosci 24: 1501, 2021]. doi:10.1038/s41593-021-00898-2. 34385700 PMC8823543

[18] Bale TL, Vale WW. CRF and CRF receptors: role in stress responsivity and other behaviors. Annu Rev Pharmacol Toxicol 44: 525–557, 2004. doi:10.1146/annurev.pharmtox.44.101802.121410. 14744257

[19] Dabrowska J, Hazra R, Guo JD, Dewitt S, Rainnie DG. Central CRF neurons are not created equal: phenotypic differences in CRF-containing neurons of the rat paraventricular hypothalamus and the bed nucleus of the stria terminalis. Front Neurosci 7: 156, 2013. doi:10.3389/fnins.2013.00156. 24009552 PMC3757458

[20] Henckens MJ, Deussing JM, Chen A. Region-specific roles of the corticotropin-releasing factor-urocortin system in stress. Nat Rev Neurosci 17: 636–651, 2016. doi:10.1038/nrn.2016.94. 27586075

[21] Lemos JC, Wanat MJ, Smith JS, Reyes BA, Hollon NG, Van Bockstaele EJ, Chavkin C, Phillips PE. Severe stress switches CRF action in the nucleus accumbens from appetitive to aversive. Nature 490: 402–406, 2012. doi:10.1038/nature11436. 22992525 PMC3475726

[22] Merali Z, McIntosh J, Anisman H. Anticipatory cues differentially provoke in vivo peptidergic and monoaminergic release at the medial prefrontal cortex. Neuropsychopharmacology 29: 1409–1418, 2004. doi:10.1038/sj.npp.1300441. 15039770

[23] Chen YW, Rada PV, Bützler BP, Leibowitz SF, Hoebel BG. Corticotropin-releasing factor in the nucleus accumbens shell induces swim depression, anxiety, and anhedonia along with changes in local dopamine/acetylcholine balance. Neuroscience 206: 155–166, 2012. doi:10.1016/j.neuroscience.2011.12.009. 22245501

[24] Lim MM, Liu Y, Ryabinin AE, Bai Y, Wang Z, Young LJ. CRF receptors in the nucleus accumbens modulate partner preference in prairie voles. Horm Behav 51: 508–515, 2007. doi:10.1016/j.yhbeh.2007.01.006. 17320879 PMC2128037

[25] Peciña S, Schulkin J, Berridge KC. Nucleus accumbens corticotropin-releasing factor increases cue-triggered motivation for sucrose reward: paradoxical positive incentive effects in stress? BMC Biol 4: 8, 2006. doi:10.1186/1741-7007-4-8. 16613600 PMC1459217

[26] Lemos JC, Shin JH, Alvarez VA. Striatal cholinergic interneurons are a novel target of corticotropin releasing factor. J Neurosci 39: 5647–5661, 2019. doi:10.1523/JNEUROSCI.0479-19.2019. 31109960 PMC6636075

[27] Bassareo V, Cucca F, Musio P, Lecca D, Frau R, Di Chiara G. Nucleus accumbens shell and core dopamine responsiveness to sucrose in rats: role of response contingency and discriminative/conditioned cues. Eur J Neurosci 41: 802–809, 2015. doi:10.1111/ejn.12839. 25645148

[28] Cheng CN, Wei Huang AC, Wu SJ. Roles of nucleus accumbens shell and core in footshock-induced stress altering behavioral sensitization by methamphetamine in acquisition and testing: Running head: stress, nucleus accumbens, and behavioral sensitization. Behav Brain Res 380: 112434, 2020. doi:10.1016/j.bbr.2019.112434. 31846629

[29] Dutta S, Beaver J, Halcomb CJ, Jasnow AM. Dissociable roles of the nucleus accumbens core and shell subregions in the expression and extinction of conditioned fear. Neurobiol Stress 15: 100365, 2021. doi:10.1016/j.ynstr.2021.100365. 34355048 PMC8319794

[30] Feja M, Hayn L, Koch M. Nucleus accumbens core and shell inactivation differentially affects impulsive behaviours in rats. Prog Neuropsychopharmacol Biol Psychiatry 54: 31–42, 2014. doi:10.1016/j.pnpbp.2014.04.012. 24810333

[31] Shin JH, Adrover MF, Alvarez VA. Distinctive modulation of dopamine release in the nucleus accumbens shell mediated by dopamine and acetylcholine receptors. J Neurosci 37: 11166–11180, 2017. doi:10.1523/JNEUROSCI.0596-17.2017. 29030431 PMC5688525

[32] West EA, Carelli RM. Nucleus accumbens core and shell differentially encode reward-associated cues after reinforcer devaluation. J Neurosci 36: 1128–1139, 2016. doi:10.1523/JNEUROSCI.2976-15.2016. 26818502 PMC4728721

[33] Xia X, Fan L, Cheng C, Eickhoff SB, Chen J, Li H, Jiang T. Multimodal connectivity-based parcellation reveals a shell-core dichotomy of the human nucleus accumbens. Hum Brain Mapp 38: 3878–3898, 2017. doi:10.1002/hbm.23636. 28548226 PMC5685173

[34] Zahm DS. Functional-anatomical implications of the nucleus accumbens core and shell subterritories. Ann N Y Acad Sci 877: 113–128, 1999. doi:10.1111/j.1749-6632.1999.tb09264.x. 10415646

[35] Cora MC, Kooistra L, Travlos G. Vaginal cytology of the laboratory rat and mouse: review and criteria for the staging of the estrous cycle using stained vaginal smears. Toxicol Pathol 43: 776–793, 2015. doi:10.1177/0192623315570339. 25739587 PMC11504324

[36] Alonso-Caraballo Y, Ferrario CR. Effects of the estrous cycle and ovarian hormones on cue-triggered motivation and intrinsic excitability of medium spiny neurons in the nucleus accumbens core of female rats. Horm Behav 116: 104583, 2019. doi:10.1016/j.yhbeh.2019.104583. 31454509 PMC7256930

[37] Land BB, Bruchas MR, Lemos JC, Xu M, Melief EJ, Chavkin C. The dysphoric component of stress is encoded by activation of the dynorphin κ-opioid system. J Neurosci 28: 407–414, 2008. doi:10.1523/JNEUROSCI.4458-07.2008. 18184783 PMC2612708

[38] van de Velden M, Iodice D'Enza A, Markos A. Distance-based clustering of mixed data. WIREs Comput Stats 11: e1456, 2018. doi:10.1002/wics.1456.

[39] Holahan MR, Kalin NH, Kelley AE. Microinfusion of corticotropin-releasing factor into the nucleus accumbens shell results in increased behavioral arousal and oral motor activity. Psychopharmacology (Berl) 130: 189–196, 1997. doi:10.1007/s002130050228. 9106918

[40] Riegel AC, Williams JT. CRF facilitates calcium release from intracellular stores in midbrain dopamine neurons. Neuron 57: 559–570, 2008. doi:10.1016/j.neuron.2007.12.029. 18304485 PMC2696265

[41] Itoga CA, Chen Y, Fateri C, Echeverry PA, Lai JM, Delgado J, Badhon S, Short A, Baram TZ, Xu X. New viral-genetic mapping uncovers an enrichment of corticotropin-releasing hormone-expressing neuronal inputs to the nucleus accumbens from stress-related brain regions. J Comp Neurol 527: 2474–2487, 2019. doi:10.1002/cne.24676. 30861133 PMC6688927

[42] Razidlo JA, Fausner SML, Ingebretson AE, Wang LC, Petersen CM, Mirza S, Swank IN, Alvarez VA, Lemos JC. Chronic loss of muscarinic M5 receptor function manifests disparate impairments in exploratory behavior in male and female mice despite common dopamine regulation. J Neurosci 42: 6917–6930, 2022. doi:10.1523/JNEUROSCI.1424-21.2022. 35896424 PMC9463982

[43] Wilson CJ, Goldberg JA. Origin of the slow afterhyperpolarization and slow rhythmic bursting in striatal cholinergic interneurons. J Neurophysiol 95: 196–204, 2006. doi:10.1152/jn.00630.2005. 16162828

[44] Arroyo A, Kim BS, Biehl A, Yeh J, Bett GC. Expression of kv4.3 voltage-gated potassium channels in rat gonadotrophin-releasing hormone (GnRH) neurons during the estrous cycle. Reprod Sci 18: 136–144, 2011. doi:10.1177/1933719110382306. 20861393

[45] Power EM, Iremonger KJ. Plasticity of intrinsic excitability across the estrous cycle in hypothalamic CRH neurons. Sci Rep 11: 16700, 2021. doi:10.1038/s41598-021-96341-4. 34404890 PMC8371084

[46] Calipari ES, Juarez B, Morel C, Walker DM, Cahill ME, Ribeiro E, Roman-Ortiz C, Ramakrishnan C, Deisseroth K, Han MH, Nestler EJ. Dopaminergic dynamics underlying sex-specific cocaine reward. Nat Commun 8: 13877, 2017. doi:10.1038/ncomms13877. 28072417 PMC5234081

[47] Shanley MR, Miura Y, Guevara CA, Onoichenco A, Kore R, Ustundag E, Darwish R, Renzoni L, Urbaez A, Blicker E, Seidenberg A, Milner TA, Friedman AK. Estrous cycle mediates midbrain neuron excitability altering social behavior upon stress. J Neurosci 43: 736–748, 2023. doi:10.1523/JNEUROSCI.1504-22.2022. 36549906 PMC9899085

[48] Clemens AM, Lenschow C, Beed P, Li L, Sammons R, Naumann RK, Wang H, Schmitz D, Brecht M. Estrus-cycle regulation of cortical inhibition. Curr Biol 29: 605–615.e6, 2019. doi:10.1016/j.cub.2019.01.045. 30744972

[49] Nappi RE, Rivest S. Ovulatory cycle influences the stimulatory effect of stress on the expression of corticotropin-releasing factor receptor messenger ribonucleic acid in the paraventricular nucleus of the female rat hypothalamus. Endocrinology 136: 4073–4083, 1995. doi:10.1210/endo.136.9.7649116. 7649116

[50] Wiersielis KR, Wicks B, Simko H, Cohen SR, Khantsis S, Baksh N, Waxler DE, Bangasser DA. Sex differences in corticotropin releasing factor-evoked behavior and activated networks. Psychoneuroendocrinology 73: 204–216, 2016. doi:10.1016/j.psyneuen.2016.07.007. 27521739 PMC5048569

[51] Davis BA, Clinton SM, Akil H, Becker JB. The effects of novelty-seeking phenotypes and sex differences on acquisition of cocaine self-administration in selectively bred High-Responder and Low-Responder rats. Pharmacol Biochem Behav 90: 331–338, 2008. doi:10.1016/j.pbb.2008.03.008. 18445506 PMC2474787

[52] Sell SL, Dillon AM, Cunningham KA, Thomas ML. Estrous cycle influence on individual differences in the response to novelty and cocaine in female rats. Behav Brain Res 161: 69–74, 2005. doi:10.1016/j.bbr.2005.01.004. 15904711

